# A simple crowdsourced delay-based traffic signal control

**DOI:** 10.1371/journal.pone.0230598

**Published:** 2020-04-07

**Authors:** Vinayak Dixit, Divya Jayakumar Nair, Sai Chand, Michael W. Levin

**Affiliations:** 1 School of Civil and Environmental Engineering, University of New South Wales, Sydney, New South Wales, Australia; 2 Department of Civil, Environmental, and Geo- Engineering, University of Minnesota, Minneapolis, Minnesota, United States of America; Huazhong University of Science and Technology, CHINA

## Abstract

Current transportation management systems rely on physical sensors that use traffic volume and queue-lengths. These physical sensors incur significant capital and maintenance costs. The ubiquity of mobile devices has made possible access to accurate and cheap traffic delay data. However, current traffic signal control algorithms do not accommodate the use of such data. In this paper, we propose a novel parsimonious model to utilize real-time crowdsourced delay data for traffic signal management. We demonstrate the versatility and effectiveness of the data and the proposed model on seven different intersections across three cities and two countries. This signal system provides an opportunity to leapfrog from physical sensors to low-cost, reliable crowdsourced data.

## Introduction

Congestion has literally put our cities at the “crossroads.” Signals are a common strategy to manage traffic at junctions through prioritization of traffic movements while ensuring the efficient and safe flow of traffic. Ever since the inception of traffic signals to manage conflicts at traffic junctions, increasing congestion has driven the pursuit towards optimal phase structure, cycle length and green times that would minimize delay and/or maximize throughput.

Traditionally, the high cost and limited access to delay data meant that most adaptive traffic signal systems relied on volume and queue length data. Notable systems of this type include SCOOT [1]; SCATS [2]; OPAC [3]; and RHODES [4]. These adaptive traffic signal systems and others that are widely used rely on physical sensors, such as loop detectors, radar sensors or near real-time traffic videos. A more recent and novel algorithms such as max-pressure [5, 6] used for adaptive signal systems at a network level also rely on queue length and volume counts that require physical sensors. Historically, the cost of acquiring volume and queue data at junctions was much more cost effective than acquiring delay data. For this reason traffic signals have not developed or utilized delay data let alone crowsourced delay data in their optimization algorithms.

The ubiquity of mobile devices has made possible each of these devices acting as a mobile sensor providing real-time information on travel time, speed, location, and traffic states. Navigation companies like Google, Here, and TomTom extensively utilize these forms of data collected from the crowd to provide real-time traffic information back to their customers. These types of data, also called “crowdsourced data” have been used to understand mobility behavior [7, 8] and congestion patterns [9]. The ubiquity and accuracy [9] of this type of crowdsourced data has made this high-quality real-time traffic data easily accessible at a substantially cheaper cost. In comparison, physical sensors still relied upon for traffic data incur significant installation and maintenance costs [10].

Despite the advances in data availability, recent work on adaptive signal control nevertheless has relied heavily on queue length information. A major topic of recent research is modifying backpressure control for communications networks [11] into an adaptive traffic signal timing [5, 12]. The main benefit of max-pressure control is the analytical *maximum-stability* property despite the decentralized control policy. Given a traffic network with stochastic demand and a specific signal control policy, the network is *stable* if the average number of vehicles in the system over time remains bounded in expectation. An unstable network indicates that the available capacity (which is partially determined by the signal timing) is insufficient for the average demand. The maximum stability property means that max-pressure control will stabilize the number of vehicles in the network if any signal control policy could stabilize the network. Extensions to max-pressure signal control have included policies for a cycle-based phase structure [6], unknown routing proportions [13, 14], realistic finite queue buffers [15, 16], and adaptive route guidance [17, 18]. Previous work has exclusively relied on queue length information for the signal control, with the exception of [19] which uses delay information from the head-of-line vehicle in each queue. The objective of this paper is quite similar. However, unlike previous studies on max-pressure control which have mostly focused on analytical models and results, we present an implementation and evaluation on public roads.

The contributions of this paper are as follows. We propose a simple, parsimonious theoretical model using crowdsourced delay for an adaptive traffic signal system. The control policy and model are directly based on max-pressure control, but have been modified to suit the practical constraints of implementation. We discuss the analytical justification for using crowdsourced delay to choose signal timings. Then, we describe how to implement max-pressure control in practice, which has yet to be accomplished in the literature. We demonstrate the use of these data and the versatility of the model for real-time signal optimization at seven different intersections across three cities and two countries. This adaptive traffic signal system provides an opportunity for developing countries with widespread mobile devices to leapfrog from physical sensors to crowdsourced data for traffic management.

## Signal control

Consider a traffic network G=(N,A) with junctions N and links A. The set of links can be divided into internal links connecting junctions, $A_{o}$, and source links where vehicles enter, $A_{z}$. Consider discretized time with time step Δ*t*. In this model, each time step represents one signal cycle, i.e. Δ*t* = *C* where *C* is the length of the cycle. Let *x*_*ij*_(*t*) be the queue length for turning movement (*i*, *j*), i.e. the number of vehicles waiting to turn from link *i* to link *j*. The evolution of queue lengths can be defined using conservation of flow. For internal links,

$$\begin{array}{c} x_{jk}\left(t+1\right)=x_{jk}\left(t\right)-y_{jk}\left(t\right)+\sum_{i\in A}y_{jk}\left(t\right)p_{ij}\left(t\right) \end{array}$$ (1)

where *y*_*ij*_(*t*) is the number of vehicles turning from *i* to *j* at time *t*, and *p*_*jk*_(*t*) is the proportion of vehicles entering link *j* turning to link *k* at time *t*. We assume that *p*_*jk*_(*t*) are independent identically distributed random variables with mean ${\bar{p}}_{jk}$. For source links, the entering vehicles are the demand *d*_*i*_(*t*), which yields

$$\begin{array}{c} x_{ij}\left(t+1\right)=x_{ij}\left(t\right)-y_{ij}\left(t\right)+d_{i}\left(t\right)p_{ij}\left(t\right) \end{array}$$ (2)

We also assume that *d*_*i*_(*t*) are independent identically distributed random variables with mean ${\bar{d}}_{i}$. The signal timing creates a boundary condition for the traffic flow. Let *g*_*ij*_(*t*)∈[0, Δ*t*] be the amount of green time allocated to turning movement (*i*, *j*) during time step *t*. Let *Q*_*ij*_ be the capacity of turning movement (*i*, *j*). Then

$$\begin{array}{c} y_{ij}\left(t\right)=\min\{x_{ij}\left(t\right),\frac{g_{ij}\left(t\right)Q_{ij}}{\Delta t}\} \end{array}$$ (3)

In most previous work on max-pressure control [e. g. 5, 12] a single signal phase is activated in each time step. In contrast, in this model multiple phases are activated each time step, making the time step more akin to a signal cycle. This model is similar to that of [6], and is chosen for practical reasons. Although phase-based control (which does not follow a conventional signal cycle) may achieve analytical maximum-stability properties, many transportation agencies and drivers are unwilling to implement a non-cyclical signal control. Agencies are rightfully concerned that drivers may be confused by signals that “skip” phases. Consequently, this model is defined with the objective of a later implementation in practice.

Travel time and delay data is ubiquitous and easily available, however, there exists no literature that utilizes this data to assign green times based on delay. We propose a parsimonious model that only requires delay information to allocate efficient green times to the different approaches at a junction. To the best of our knowledge, though simple, it is the first time this model is proposed. Let *τ*_*ij*_(*t*) be the delay for turning movement (*i*, *j*) at time *t*. Notice that the delay does not correspond exactly to the queue lengths *x*_*ij*_(*t*). For instance, it is possible to have a queue length of one vehicle, i.e. *x*_*ij*_(*t*) = 1, yet have an arbitrarily high delay. Similarly, it is possible to have a queue length of multiple vehicles with an average delay of *τ*_*ij*_(*t*) = 1. However, these situations are likely to represent unusual realizations of stochastic demand and turning proportions, especially when the signal control is responsive to delay. In reality, *x*_*ij*_(*t*) and *τ*_*ij*_(*t*) are likely to be highly correlated. When the queue length is large, the delay is likely to be correspondingly high, and vice versa. Our objective is to relate *x*_*ij*_(*t*) to *τ*_*ij*_(*t*). To do so, we must look at the long-term scenario, which is the stability analysis of max-pressure control.

### Stability discussion

The traffic network is defined to be stable if the queue lengths remain bounded in expectation, i.e. there exists a *κ* < ∞ such that for all *T*,

$$\begin{array}{c} \frac{1}{T}\sum_{t=1}^{T}\sum_{\left(i,j\right)\in A^{2}}E[x_{ij}\left(t\right)]\leq \kappa \end{array}$$ (4)

Obviously, it is possible to create average demand rates that are unstable (the demand exceeds the capacity of the turning movement). To compare demand and capacity for internal links, we must relate entering demand (at source links) to incoming flow in internal links. Let λ_*ij*_ (vector **f**) be the average volume for link *i*. Disregarding capacity limitations induced by the signal timing, we can relate **λ** to the vector of average demand $\bar{d}$ and the vector of average turning proportions $\bar{p}$ as follows:

$$\begin{array}{ccc} \lambda_{ij}={\bar{d}}_{i}{\bar{p}}_{ij} & \;\forall i\in A_{\text{z}} & \;\left(5\text{a}\right)\\ \lambda_{jk}=\sum_{i\in A}\lambda_{ij}{\bar{p}}_{jk} & \;\forall j\in A_{\text{o}} & \;\left(5\text{b}\right) \end{array}$$

Consider a sequence of green time allocations *G* = **g**(*t*). Then the average green time allocation for *G* is

$$\begin{array}{c} {\bar{g}}_{ij}=\lim_{T\to \infty }\frac{1}{T}\sum_{t=1}^{T}g_{ij}\left(t\right) \end{array}$$ (6)

Notice that $\lambda_{ij}{\bar{p}}_{ij}$ is the average demand for turning movement (*i*, *j*). If there exists a control sequence such that

$$\begin{array}{c} \lambda_{ij}\leq \frac{{\bar{g}}_{ij}Q_{ij}}{C} \end{array}$$ (7)

for all $\left(i,j\right)\in A^{2}$, then the average demand defined by $\bar{d}$ and $\bar{p}$ is stabilizable. [5] developed an analytical max-pressure policy that can stabilize the network if Eq (7) holds with strict inequality for all turning movements.

The objective of this discussion is to relate queue lengths with delays. In an unstable network, violating constraint (7) for turning movement (*i*, *j*) prevents the incoming flows of turning movements (*j*, *k*) for k∈A from being defined by constraints (5). On the other hand, in a stable network, *f*_*i*_ defines both the average entering and exiting flow for link *i*. Let ${\bar{x}}_{ij}$ be the average queue length and let ${\bar{\tau }}_{ij}$ be the average delay for turning movement (*i*, *j*). Little’s Law [20] relates **λ**, $\bar{x}$, and $\bar{\tau }$ as follows:

$$\begin{array}{c} {\bar{x}}_{ij}=\lambda_{ij}{\bar{\tau }}_{ij} \end{array}$$ (8)

Although it is a classic result in queueing theory, it only holds for the limiting behavior. In other words, Eq (8) can only relate average queue lengths and delays, and not the specific realizations of queue lengths and delay at an arbitrary time *t*. While this restricts the analytical results that we can obtain, we nevertheless exploit the long-run relationship between queue length and delay through a new adaptive signal policy that is evaluated on public roads.

### Max-pressure control policy

Given the relationship between queue length and travel times, we propose a novel pressure-based policy for allocating green time. We do not use the policy of [19] because they choose the optimal phase per time step, whereas we must allocate green time to each phase at each time step to maintain the signal cycle structure. Consider an individual junction *n* with turning movements $A_{n}^{2}$. Observe that given a queue of *x*_*ij*_(*t*), the amount of green time allocated for an approach within a cycle that would ensure clearance of queue is

$$\begin{array}{c} x_{ij}\left(t\right)=Q_{ij}g_{ij}\left(t\right) \end{array}$$ (9)

The objective of this control policy is to replace explicit knowledge of *x*_*ij*_(*t*), which requires sensors to obtain, with crowdsourced data providing *τ*_*ij*_(*t*). Using the relationship from Eq (8) as a heuristic substitution for *x*_*ij*_(*t*), we obtain

$$\begin{array}{c} g_{ij}\left(t\right)=\frac{\lambda_{ij}\tau_{ij}\left(t\right)}{Q_{ij}} \end{array}$$ (10)

Eq (10) specifies the green time per movement. However, signal control is processed via phases. Let $S_{n}$ be the ordered set of phases for junction *n*, with *C*_*n*_ the cycle length and *L*_*n*_ the lost time. Each phase $s\in S_{n}$ proceeds in a specified order, but the green time *g*_*s*_(*t*) allocated to each phase can vary. Define $s\subset A^{2}$ to be a subset of turning movements that are activated during the phase. We modify Eq (10) to specify the green time per phase, *g*_*s*_(*t*), as follows:

$$\begin{array}{c} g_{s}\left(t\right)=\max_{(i,j)\in s}\{g_{ij}\left(t\right)\}=\max_{(i,j)\in s}\{\frac{\lambda_{ij}\tau_{ij}\left(t\right)}{Q_{ij}}\} \end{array}$$ (11)

Eq (11) indicates a pressure (based on delay) for allocating green time to phase *s*. This relationship must be refined because the quantity of available green time per cycle is limited, and the specified green time of Eq (11) may exceed those limits. We use the pressure in Eq (11) to allocate the available green time proportionally. Define *Λ*_*n*_ to be a common maximum arrival rate for junction *n*:

$$\begin{array}{c} \Lambda_{n}=\max_{ij\in A_{n}^{2}}\{\lambda_{\left(i,j\right)}\} \end{array}$$ (12)

We make this substitution because knowing the exact arrival rates would require sensors to measure flow. Using Eq (12) in conjunction with Eq (11), we obtain

$$\begin{array}{c} g_{s}\left(t\right)=\max_{(i,j)\in s}\{\frac{\Lambda_{n}\tau_{ij}\left(t\right)}{Q_{ij}}\} \end{array}$$ (13)

which is based on the maximum delay for any movement (*i*, *j*)∈*s*. For a fixed cycle length the total green time is constrained by

$$\begin{array}{c} \sum_{\left(i,j\right)\in A_{n}^{2}}=C_{n}-L_{n} \end{array}$$ (14)

Combining Eqs (13) and (14), we obtain

$$\begin{array}{c} \Lambda_{n}=\frac{C_{n}-L_{n}}{\sum_{s\in S_{n}}\max_{(i,j)\in s}\{(\tau_{ij}\left(t\right)/Q_{ij})\}} \end{array}$$ (15)

Therefore, we allocate green time as

$$\begin{array}{c} g_{s}\left(t\right)=\frac{\max_{(i,j)\in s}\{(\tau_{ij}\left(t\right)/Q_{ij})\}}{\sum_{s^{\prime }\in S_{n}}\max_{\left(i^{\prime },j^{\prime }\right)\in s^{\prime }}\{(\tau_{i^{\prime }j^{\prime }}\left(t\right)/Q_{i^{\prime }j^{\prime }})\}}(C_{n}-L_{n}) \end{array}$$ (16)

Unlike previous methods, this model only relies on delays and saturation flow rates to determine the green times that ensures that the queues are cleared. The saturation flow rates for the movement can be calibrated a-priori (or using HCM 2016), and the delay data is measured in real-time. The fact that the model does not require real-time arrival rates or queue lengths empowers traffic signal managers to avoid costly physical sensors (eg. loop detectors or cameras) that would have been required for its measurement. As commonly done in developing countries, to accommodate for mixed traffic the saturation flow rate is normalized by using passenger car units (pcu) as a standardization.

If the arrival rates to movement (*i*, *j*) are exactly λ_*ij*_, then Eq (7) indicates that the queue on (*i*, *j*) will not grow when

$$\begin{array}{c} \lambda_{ij}\leq \frac{\tau_{ij}\left(t\right)}{\sum_{\left(i^{\prime },j^{\prime }\right)\in A_{n}^{2}}(\tau_{i^{\prime }j^{\prime }}\left(t\right)/Q_{i^{\prime }j^{\prime }})}\frac{C_{n}-L_{n}}{C} \end{array}$$ (17)

Of course, stochasticity affects the realization of the arrival rates, as well as the delays. As previously discussed, relating *τ*_*ij*_(*t*) to *x*_*ij*_(*t*) at each time is challenging analytically, which makes a proof of stability difficult. Instead, we focus on conducting field experiments on public roads to validate the benefits of this signal timing policy.

## Field experiments

**Fig 1:**
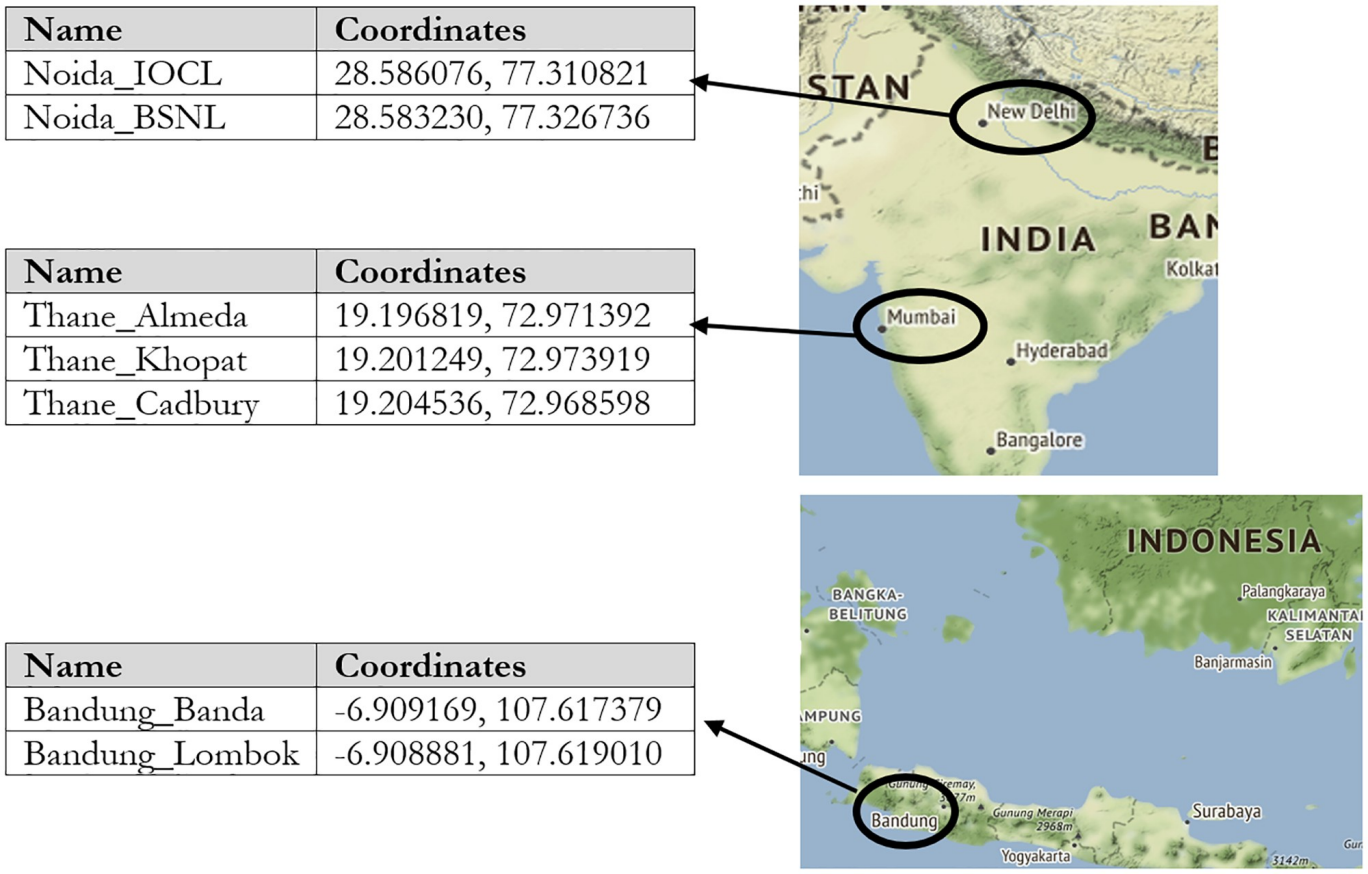
Geographical locations of the field experiments with latitude and longitudes. Image courtesy of the Earth Science and Remote Sensing Unit, NASA Johnson Space Center.

We conducted international field experiments across two different countries, at seven signalized intersections in three different cities, namely Thane (India), Bandung (Indonesia), and Noida (India). The locations of the intersections are shown in Fig 1. The purpose of the large scale international trial was to demonstrate the geographical robustness of this methodology. The real-time delay data was acquired using the Google Maps Delay API at 5 min intervals. Table 1 shows the dates the data that were collected during peak traffic conditions for the testing and non-testing periods. During the non-testing period the signals were running based on pre-determined fixed time signals.

**Table 1:** Time periods for testing and non-testing.

Signal	Dates of testing	Dates of the base period (Non-testing)	Month	Peak hours
Thane_Almeda	16–18, 21, 22	1–11, 14, 15, 23	May 2018	9AM–2PM & 4PM–9PM
Thane_Khopat	31, 1, 4, 5	18, 21, 28, 29, 6, 7	May and June 2018	9AM–2PM & 4PM–9PM
Thane_Cadbury	7	4–6, 8, 11, 12	June 2018	9AM–2PM & 4PM–9PM
Noida_BSNL	7, 8	4, 5, 6	March 2019	9AM–2PM & 4PM–9PM
Noida_IOCL	18, 19, 22, 25–28	14,15	March 2019	9AM–2PM & 4PM–9PM
Bandung_Banda	3, 6	4, 13	August 2018	7AM–12PM & 4PM–8PM
Bandung_Lombok	3, 6	4, 13	August 2018	7AM–12PM & 4PM–8PM

### Implementation

As most intersections had a similar lane configuration for all the approaches, resulting in nearly-identical saturation flow rates (*Q*_*ij*_), Eq (16) reduces to only delays:

$$\begin{array}{c} g_{ij}\left(t\right)=\frac{\tau_{ij}\left(t\right)}{\sum_{\left(i^{\prime },j^{\prime }\right)\in A_{n}^{2}}\tau_{i^{\prime }j^{\prime }}\left(t\right)}(C_{n}-L_{n}) \end{array}$$ (18)

The cycle times and signal phasing structure at each intersection were maintained in their original structure. This delay data was used to calculate the green time for each phase using Eq (18). The new green times for the phases were then executed at the intersection by the signal controller.

**Fig 2:**
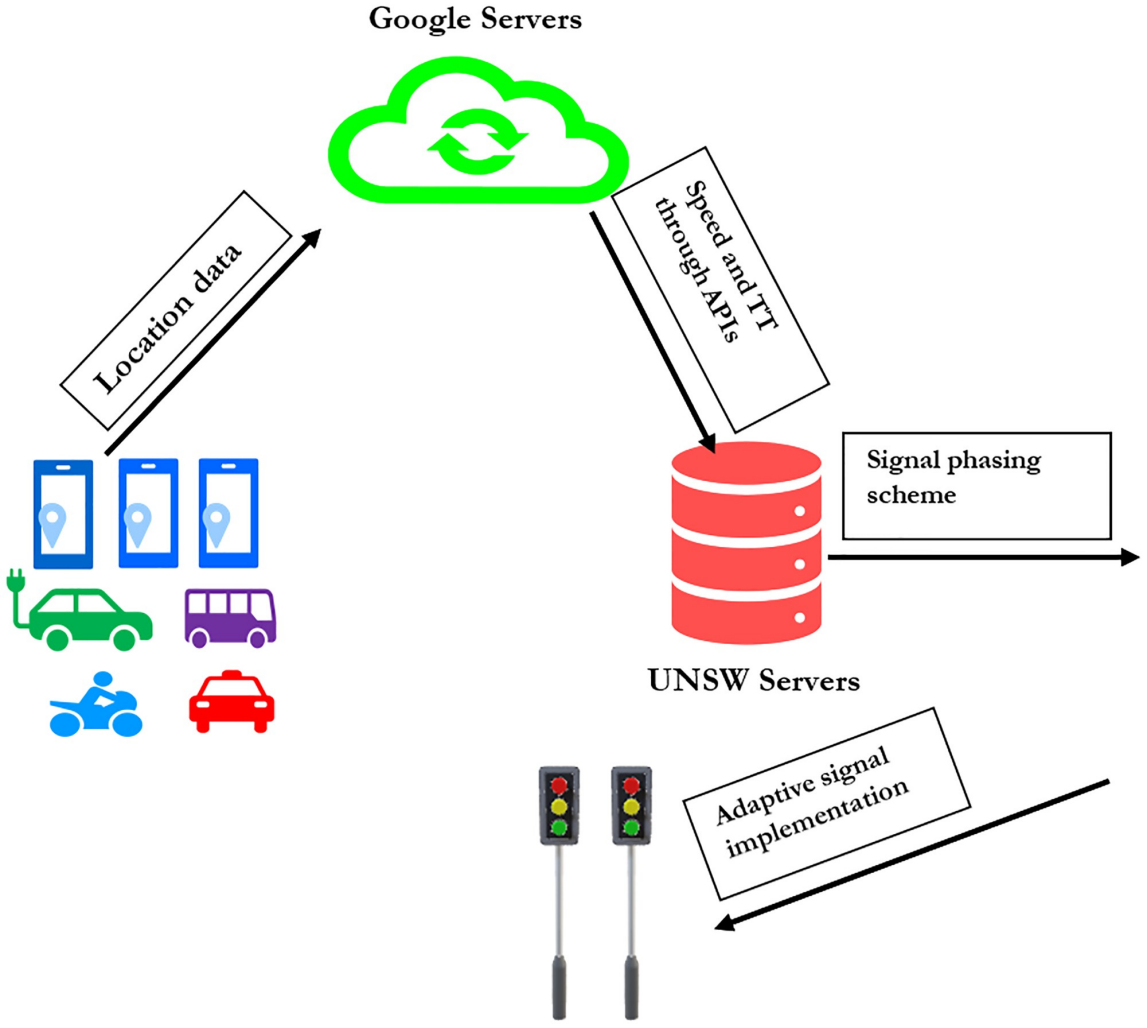
Representation of architecture for CTMS using Google data.

Arduino, a low-cost and open-source I/O motherboard controller, was installed at the intersections and it receives input from Google data (through a remote server at UNSW). The main advantage of Arduino is its ability to load the experimental script on the motherboard’s memory and let it run without interfacing with computers or external software [21]. The Arduino controller was equipped with a mobile sim card with 3G internet connectivity to be able to communicate with the servers. Fig 2 shows the architecture of Crowdsourced Traffic Management System (CTMS) using Google data.

### Results

**Fig 3:**
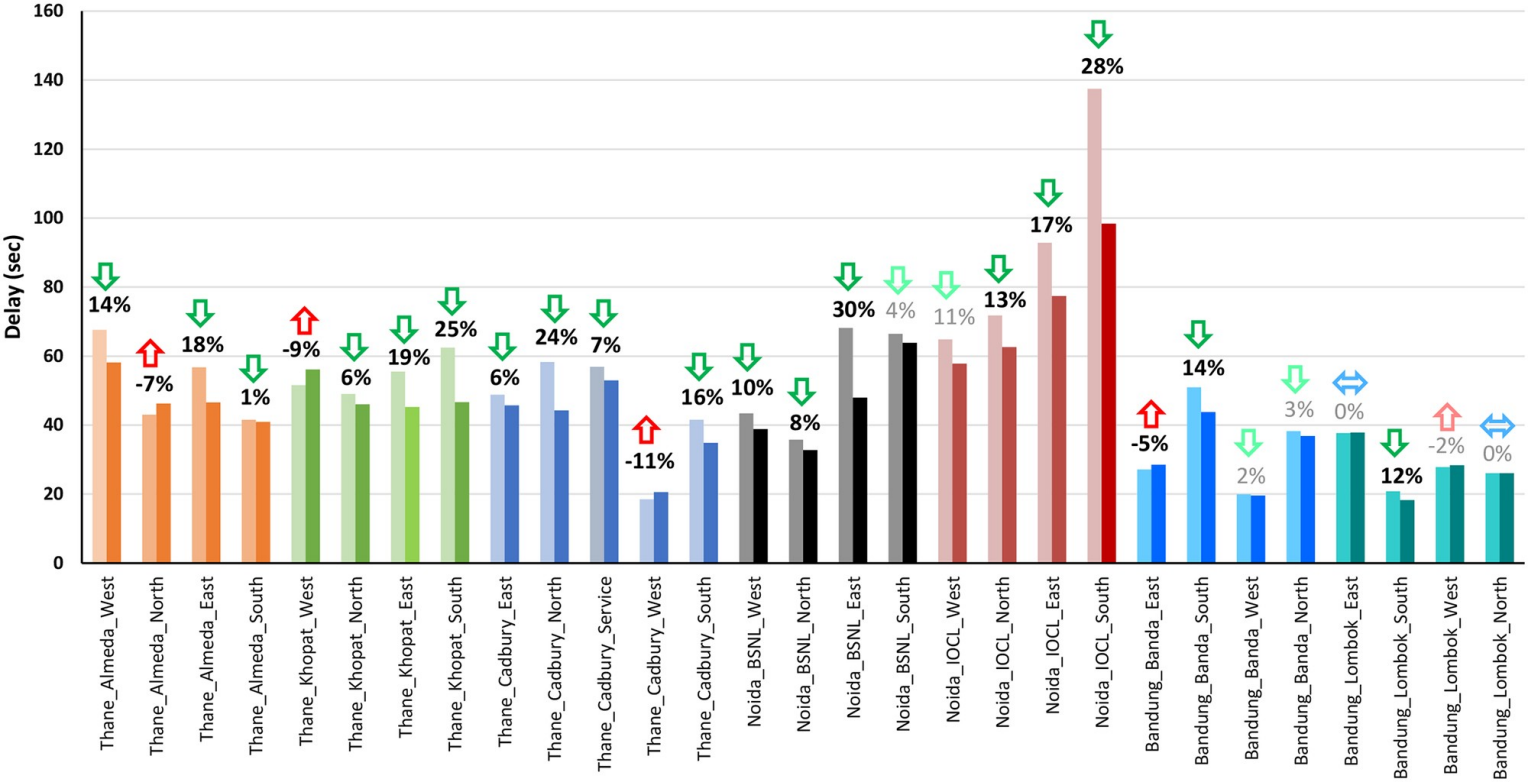
Sample delay measurements from Google Maps for one approach.

In this study, we used “approach-wise delay” to evaluate the effectiveness of the proposed crowdsourced adaptive signal system. Delay information for each movement was obtained through the Google Maps API. To evaluate the effectiveness of the proposed control strategy, a comparison was conducted between the testing and non-testing periods All data needed to replicate the findings are available at https://github.com/vvdixit/CrowdsourcedSignalEval. Fig 3 shows a sample of the observed delay measurements for one approach at an intersection. The sharp and frequent variations show significant responsiveness to real-time vehicle delays, which should be positively correlated with queue lengths. Therefore, Fig 3 indicates that the crowd-sourced data is likely a precise real-time measurement for use within pressure-based signal timings.

**Fig 4:**
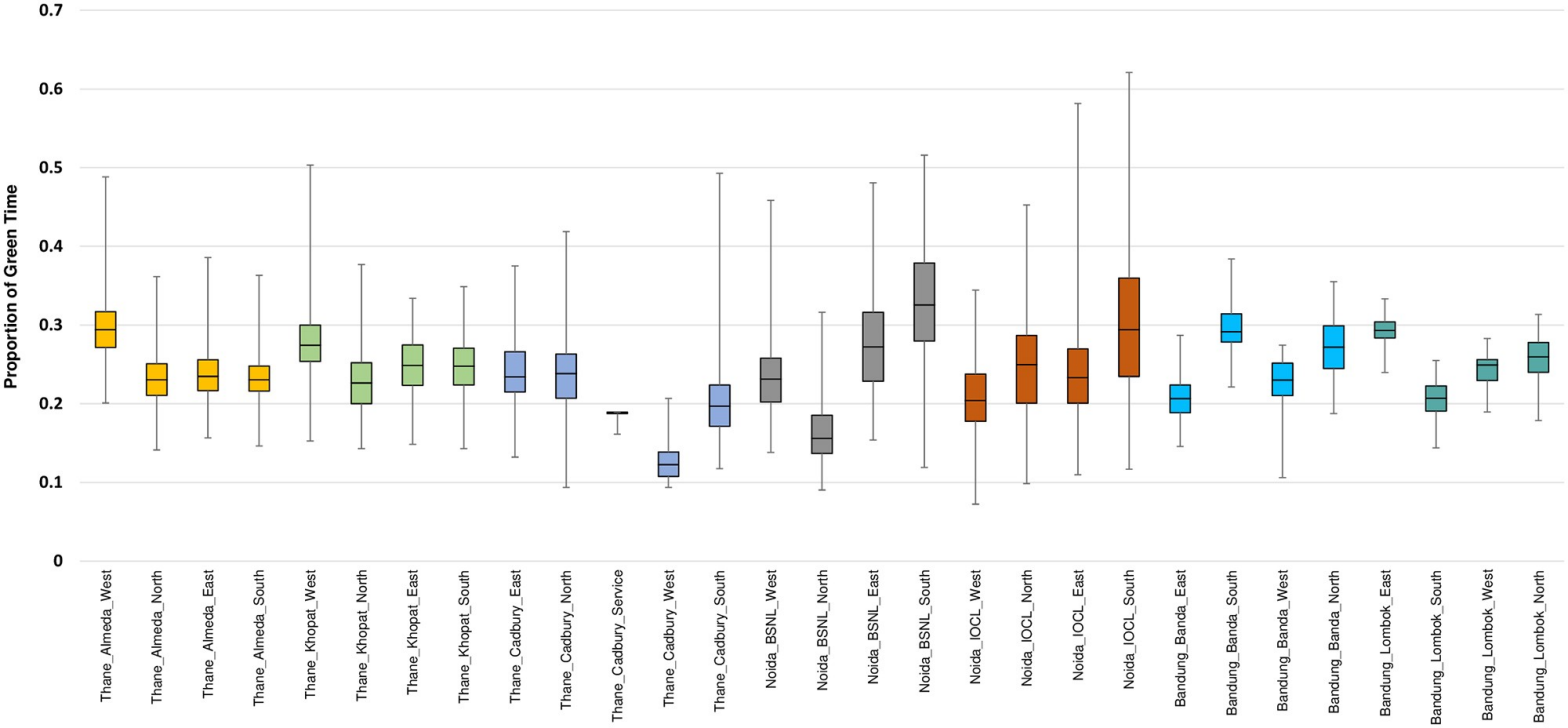
Comparison of approach-wise delay before and after.

**Fig 5:**
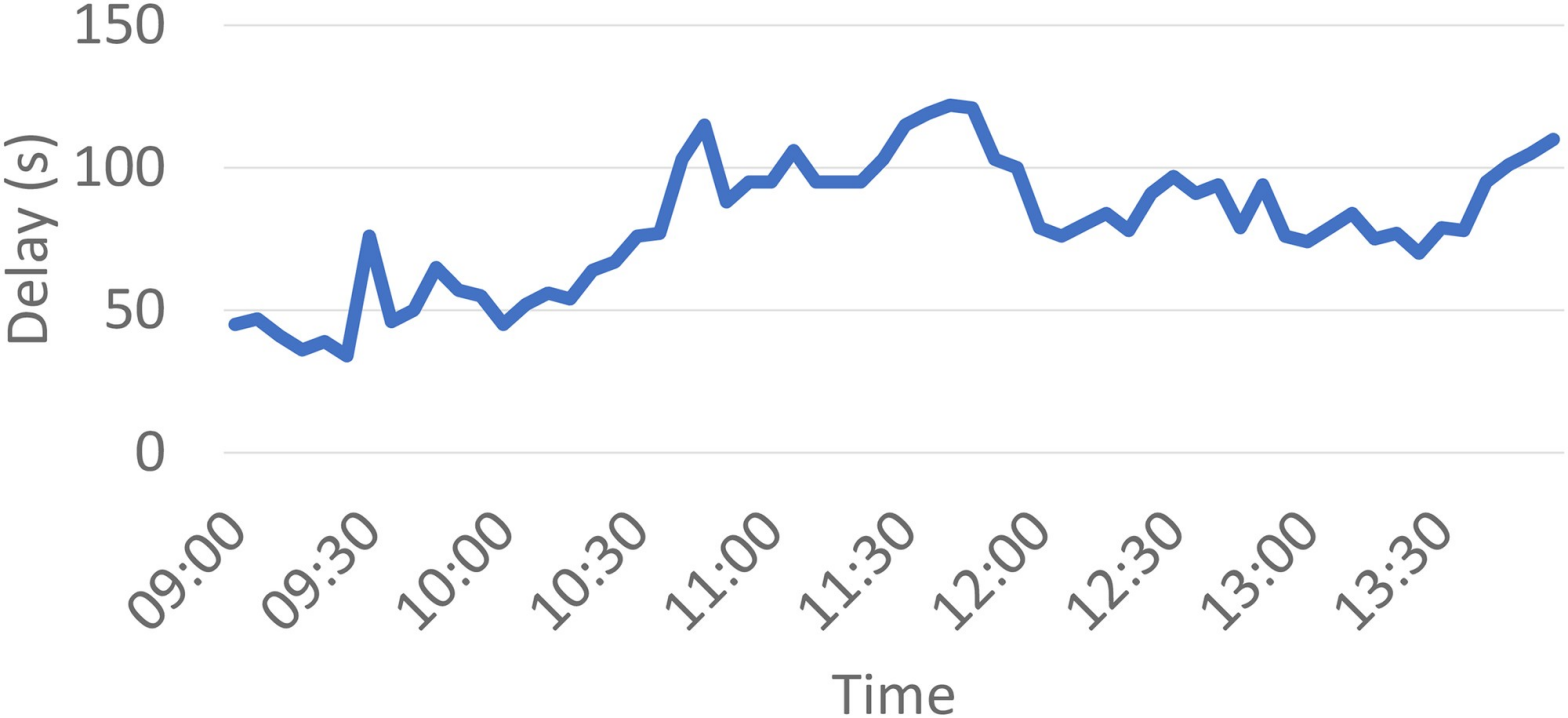
The proportion of green time for each approach.

Fig 4 summarizes the results in terms of delays and percentage reduction in delays during peak hours, which were identified using the approach outlined in [9]. Every intersection was found to have a significant reduction in delays on the majority of the approaches. A *t*-test was conducted and statistically significant reductions were observed at a 95% confidence and are shown in bold in Fig 4. Changes in delay that were not found significant are shown in grey. The maximum reduction in approach delays across all intersections ranged from 12–30%. Although not surprising, some approaches were found to have a marginal increase in delays; these were typically the minor approaches with less traffic that got allocated shorter green times. For the approaches where delay increased significantly, other approaches at the same intersection experienced larger reductions in delay. This indicates that although some approaches were penalized by this new signal timing, the intersection experienced overall benefits. The proportion of green of the cycle time provided to each approach is shown in Fig 5. The values shown are aggregated over all of the controlled cycles.

## Discussion

Inspired by max-pressure signal control, this study proposes a novel model to allocate the length of green time to a phase using real-time crowdsourced delay data. The novelty of this parsimonious model is that it only relies on real-time delay data and not requiring any real-time traffic volume or queue length data. Most adaptive signal controls, including most previous work on max-pressure control, rely on queue lengths, which require installation and frequent maintenance of multiple loop detectors or video detectors at each intersection. Furthermore, physical sensors are well known to not work properly in mixed modes and shared lane traffic. The movement based crowdsourced delay data acquired through these platforms provide actual experienced delays in such contexts. This paper demonstrates an effective pressure-based signal control that uses crowdsourced delay data, which can be obtained at much lower cost. We demonstrate the versatility and effectiveness of this approach across three cities in two different developing countries. This approach provides a cost-effective solution to leapfrog from physical sensors to ubiquitous crowdsourced data and disrupt traditional signal systems. The Arduino controllers used are quite cheap (roughly US$35) and were a low-cost alternative for loop detectors and other sensors which are far more expensive to install and operate.

Future work should advance the practical utility of max-pressure control through several open issues. First, most previous studies on max-pressure control have ignored the signal cycle structure of phase timing, which practitioners are reluctant to abandon. Analytical work on max-pressure control based on crowdsourced delay, as opposed to measured queue lengths, could be more widely implemented without the costly reliance on sensor installation and maintenance.

## Supporting information

S1 File (XLSX)

S2 File (XLSX)
